# X-ray computed tomography (CT) and ESEM-EDS investigations of unusual subfossilized juniper cones

**DOI:** 10.1038/s41598-021-01789-z

**Published:** 2021-11-16

**Authors:** Wafaa A. Mohamed, Maisa M. A. Mansour, Mohamed Z. M. Salem, Hayssam M. Ali, Martin Böhm

**Affiliations:** 1 Conservation Department, Faculty of Archaeology, Cairo University, Giza, 12613 Egypt; 2 Forestry and Wood Technology Department, Faculty of Agriculture (EL-Shatby), Alexandria University, Alexandria, 21545 Egypt; 3 Botany and Microbiology Department, College of Science, King Saud University, P.O. Box 2455, Riyadh, 11451 Saudi Arabia; 4 Department of Materials Engineering and Chemistry, Faculty of Civil Engineering, Czech Technical University in Prague, Thákurova 7, 166 29, Prague 6, Czech Republic

**Keywords:** Ecology, Evolution, Plant sciences

## Abstract

Recent investigations of a Greco-Roman site at Sais have provided well-preserved archaeobotanical remains within a pile of metal fragments. The remains are compared with comparable modern taxa. The morphology and anatomy are studied using Light microscope (LM), Environmental scanning electron microscope (ESEM) and X-ray computed tomography (CT). To investigate the preservation mode, Energy dispersive spectroscopy (EDS) analysis and elemental mapping are conducted. Results revealed that the archaeobotanical remains are exhibiting close affinity with modern juniper cones. Although, the studied archaeobotanical remains are buried for more than 2 millenniums, they underwent early stages of silicification and copper mineralization. These results are discussed in relation to other excavated objects in the find and to our knowledge and understanding of daily life in the Greco-Roman period.

## Introduction

During the 1990s discoveries of the Supreme Council of Antiquities (SCA) red brick buildings, a Hellenistic- Roman bath-house and a Hellenistic-Roman bronze hoard were excavated in Sais (Sa el-Hagar). Sais was the capital of Egypt in the 26th Dynasty (664–525 BC). The bronze hoard contained some famous statuettes of Aphrodite, a statuette of wrestlers and a pile of metal fragments in a bucket. An approach for the virtual reconstruction of the fragments identified the original objects as mostly utensils^1^, Within which, well preserved botanical remains were found (Figs. 2a–c). This was surprising as plant remains are found only in places where decay is inhibited^2^. The four most common modes of preservation encountered in archaeobotany, are charring or carbonization, water logging, desiccation, and mineralization (mineral replacement). The actual mode of preservation matters greatly, because each type of preservation preserves a slightly different range of plant types^3^.

The Sais settlement was constructed in the Nile Delta^4^, (30°58′05ʺ N, 30°45′56ʺ E), 1.8 km east of the Rosetta Branch. The site covers an area of 0.16 km^2^ and elevated 5–6 m above mean sea level^5^. Since the late Holocene period till the present, the Nile delta margin has experienced submergence^4,6–8^. The soil of the site is silty clay^4,5^, and thus the pores are too small and very low oxygen levels exist because of the constant dampness; this provides anaerobic, or almost anaerobic, conditions. These conditions can be preserving^9,10^. In such environment organic decomposers become inactive. Therefore, the remaining organic materials are possibly preserved. Metals if present in the soil, such as copper, silver, and iron, they oxidize, creating metal oxides that are very toxic to bacteria and fungi, increasing preservation possibilities^11^. Other conditions such as waterlogged, arid, low-energy environments or sometimes frozen environments can also be preserving for organic remains^2,12,13^. Desiccation takes place in dry environments like desert or in dry sheltered places like houses^14^, or when moisture levels are too low for organic decomposition to occur^15^.

Preserved archaeobotanicals are very important as they give information about everyday activities, which were rarely discussed in surviving texts, about the practice of producing food, the daily chore of preparing the food and disposing the waste, about the eating habits, about nutrition and health, about social status and character and generally about the crucial role of plants in personal lives^3^.

This study aims to identify the excavated archaeobotanical material, illuminate the environmental and archaeological context, to increase our knowledge about its importance and use. The study also aims to explain the specific preservation mode and to find out the impact of the presence of metal fragments on the preservation mode.

## Material and Method

### Sample collection

**Figure 1:**
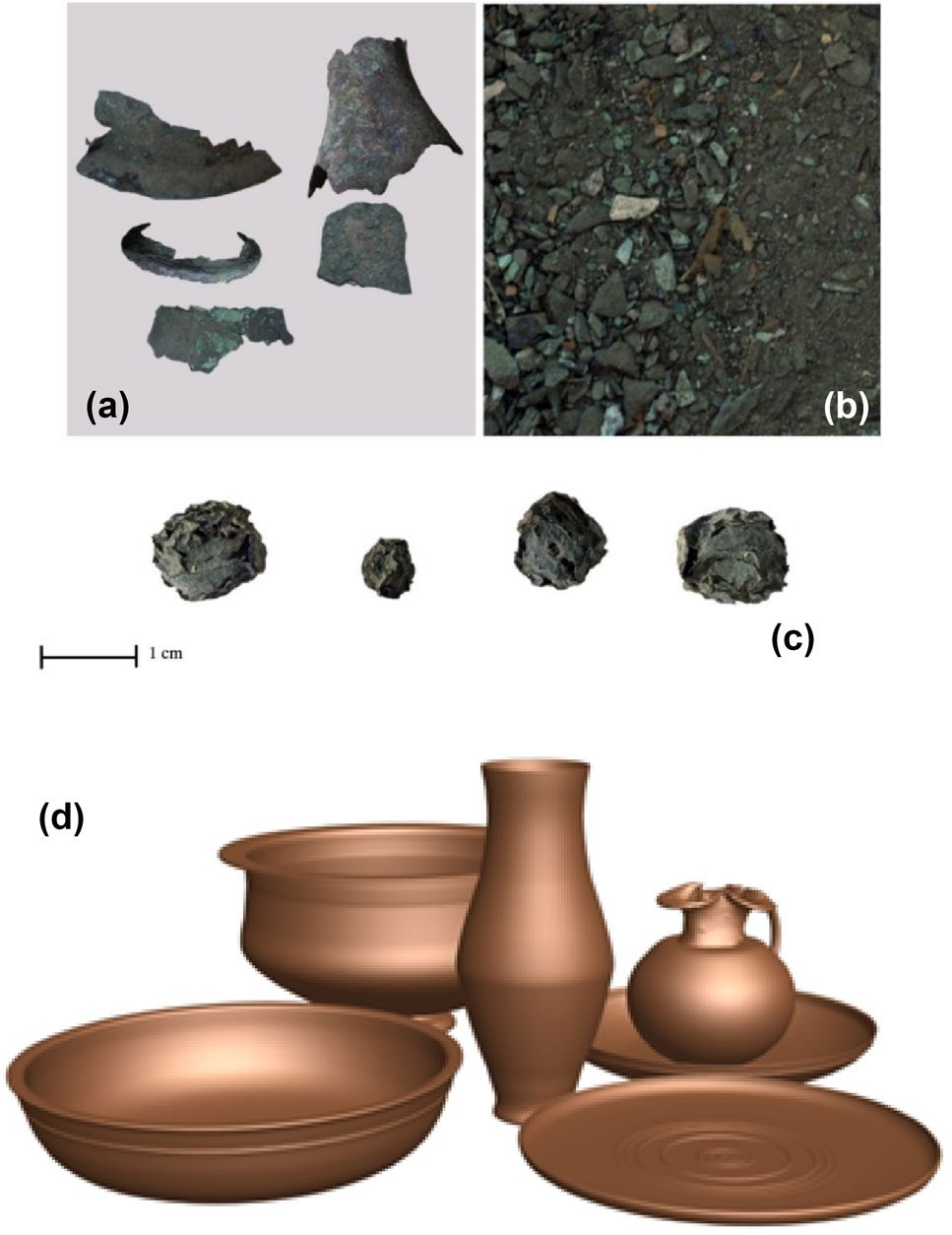
The excavated metal fragments showing variable shapes and sizes (**a**), other tiny fragments in a mixture of corrosion products and soil grains (**b**), the archaeobotanical material (**c**) and utensils rendered by virtual reconstruction of fragments (**d**)^1^ (Processed using Adobe Photoshop CC2019).

This study is complied with relevant institutional, national, and international guidelines and legislation. This study does not contain any studies with human participants or animals performed by any of the authors, where archaeobotanical material and the associated metal fragments have been registered as (nos. 19–97/43/11 A-Q) (Fig. 1a–d), in Tanta Museum, Egypt.

### Light microscopy examination and SEM–EDS micromorphology

The archaeobotanical specimens were observed and photographed using (Leica EZ4) Stereo Microscope and Canon EOS 4000D/Rebel T 100 Digital SLR Camera.

Modern dried juniper cones were collected from the best-selling herbal market in Egypt (Harraz for food Industry and Natural Products, Egypt). One of them was sectioned for comparison with the archaeobotanical specimen. The modern specimen was soaked overnight in warm water then cut with a scalpel to show the number and arrangement of seeds.

For transverse sectioning, one of the archaeobotanical specimens was embedded in a polyester resin block then cut into two halves using a jeweler saw then polished using a series of coarse- to fine-grit SiC polishing paper and ground down to 2000 grit. The same procedure was conducted for sectioning the modern juniper specimen for micromorphological examination and analysis.

The transverse section micromorphology of the archaeobotanical specimen was investigated using an environmental scanning electron microscope with energy dispersive spectroscope SEM–EDS (Quanta FEG250, with tungsten electron source, at 20 kV). Four analysis spots were analyzed. Mapping was undertaken through the scanned area to show the distribution and relative proportion (intensity) of the defined elements. The "ZAF correction method" was used for quantitative analysis of elements.

### C-scanning examination

The archaeobotanical specimen was scanned using X-ray computed tomography (CT) Scanning at Majd El-Eslam Medical Centre, Egypt, using Toshiba Aquilion 16 CT Scanner, Japan. Datasets were visualized, and images and videos were captured, 3-D Imaging: image quality with surface shaded-renderings and volume-rendered 3-D images. Zooming and panning over the 3-D surface and performs distance measurements. High Image Quality: The Aquilion 16 features 896 channels in 40 rows of solid-state detectors; specialized, user-selectable, image-reconstruction algorithms; and a wide selection of slice thicknesses. The system provides low-contrast resolution of 2 mm at 0.3% and high-contrast resolution of 0.35 mm. The used parameters are as follows: voltage 120 kV, current150 mA, timing 15.819 s, no. of X-ray projection, thickness 0.5 X 16 mm.

## Results

### Morphological visualizing and CT examinations of archaeobotanical and modern specimens

**Figure 2:**
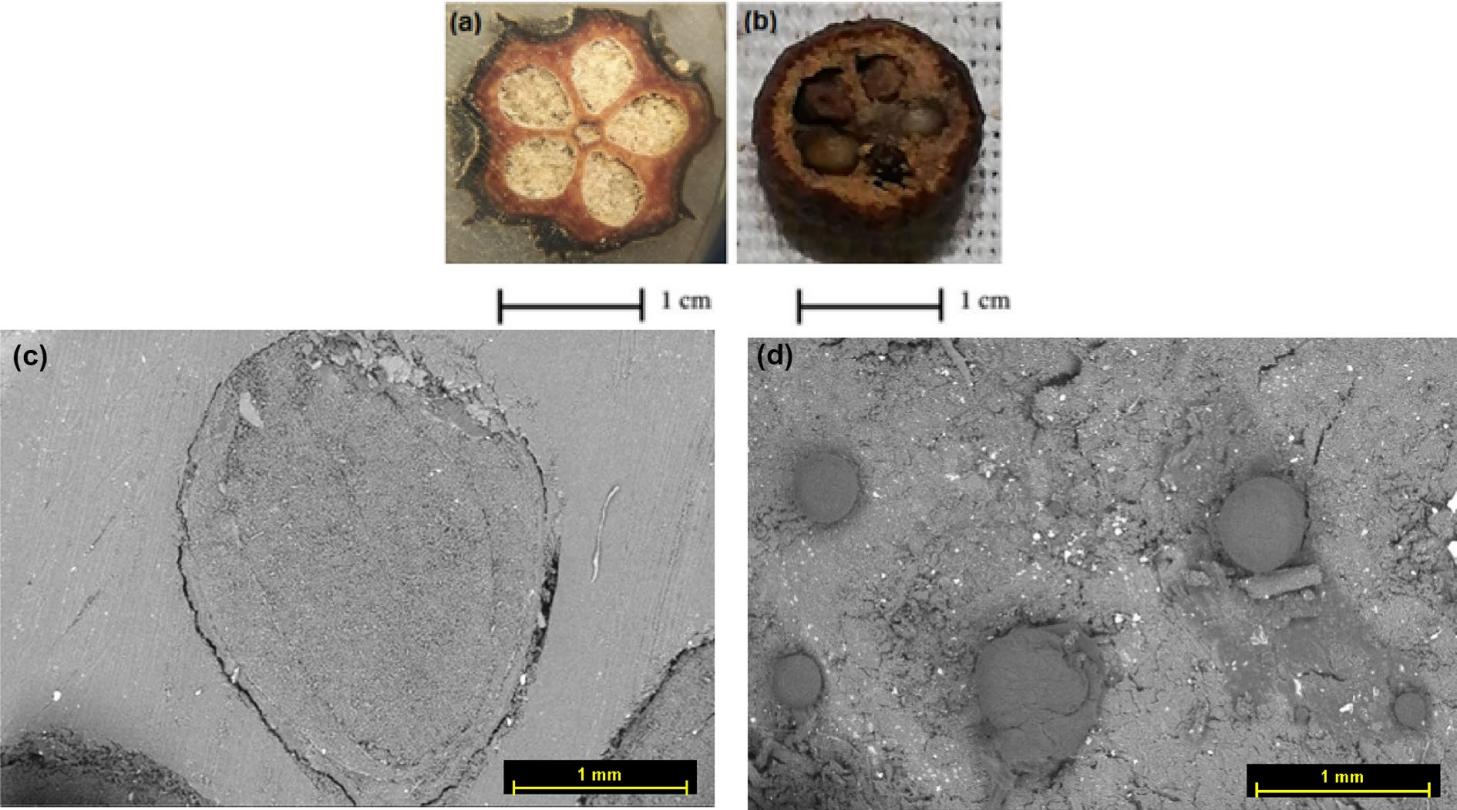
Transverse section of an archaeobotanical specimen, showing five seeds in a star arrangement, a scaley skin (**a**). The modern Juniper cone specimen showing similar characteristics (**b**). SEM image of archaeobotanical specimen showing seeds (**c**) and SEM image for modern dried juniper cone (**d**) at Scale bar = 1 mm.

Taphonomy; Visual and microscopic examination (Fig. 2a) revealed that the archaeobotanical material composed of five rounded to oval shaped, 0.8–1.3 cm diameter cone withdark brown/black surface colour with green hue. The surface was rough with scaly skin. The archaeobotanical cone specimen seemed exceptionally well-preserved when compared with modern taxa. The size, shape features and the transverse section of the archaeobotanical cone specimen show that it includes intact seeds. The transverse section found similar to modern Juniper cone specimen (Fig. 2b). At scale bar of 1 mm using SEM examination, the archaeobotanical specimen (Fig. 2c) was compared with the modern juniper specimen (Fig. 2d). The morpho-taxonomic study revealed that they are having a close affinity with each other.

**Figure 3:**
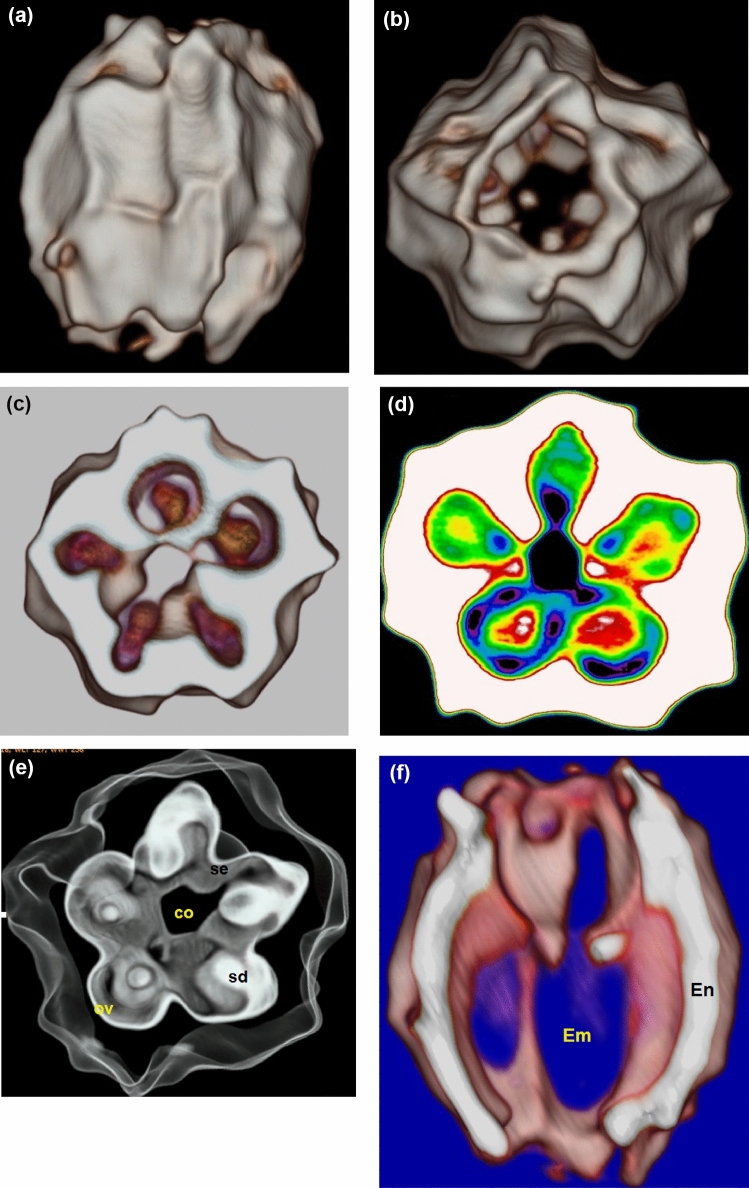
CT tomography showing the morphological features of the archaeobotanical specimen; (**a**) surface side view; (**b**) surface top view; (**c**,**d**) transverse sections showing the seeds within ovules; (**e**) scanned whole view of the cone (*Se* septa, *Co* columella, *Sd* seed, *Ov* ovules); (**f**) longitudinal or side view (*En* endosperm, *Em* embryo).

By using the CT scanner for the archaeobotanical cone specimen, the surface appearance, the rough skin (Fig. 3a) and the scaled surface (Fig. 3b) are clearly shown. Five fleshy fused scales, each with a single seed are shown with smooth layer (Fig. 3c–e). The longitudinal or side view of the cone is shown in Fig. 3f.

**Figure 4:**
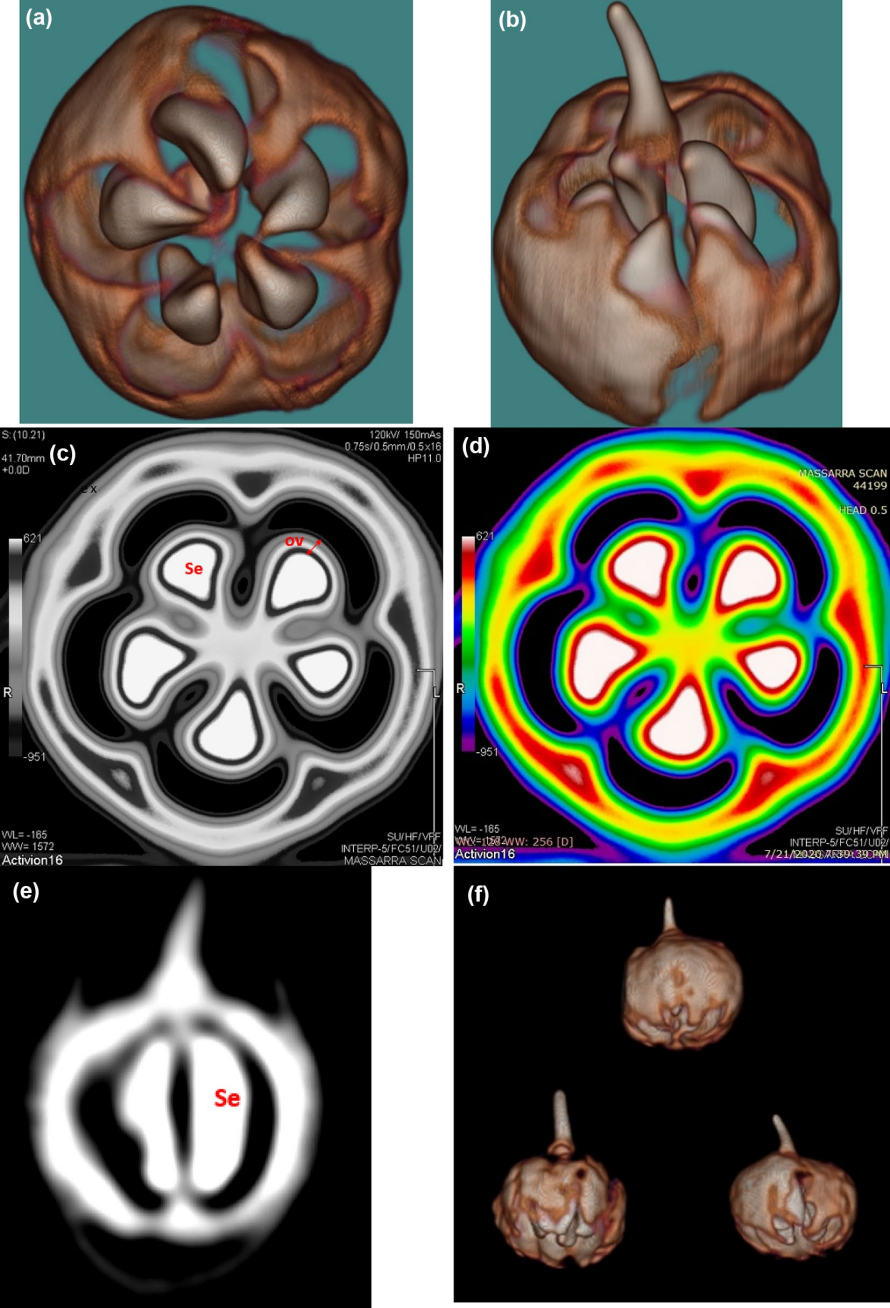
CT scanning of the modern fruit specimen; top view (**a**) and side view (**b**), transverse sections in the endocarp (**c**,**d**) *Ov* ovules; longitudinal section (*Se* seed) (**e**), and the shape and form of the whole cone (**f**).

For the modern dried cone (Fig. 4), the top and inside views are clearly shown in Fig. 4a,b, respectively, with the arrangements of five fleshy scales. In the cone transverse sections (Fig. 4c,d) the cone’s anatomical structure with five scales are clearly shown under CT scanning, each with one seed (Fig. 4e) and the longitudinal scanned view with the anatomical structure of one scale are illustrated in (Fig. 4e), while the whole shape of the cone is shown in Fig. 4f.

### Microchemical analysis of the archaeobotanical material

**Figure 5:**
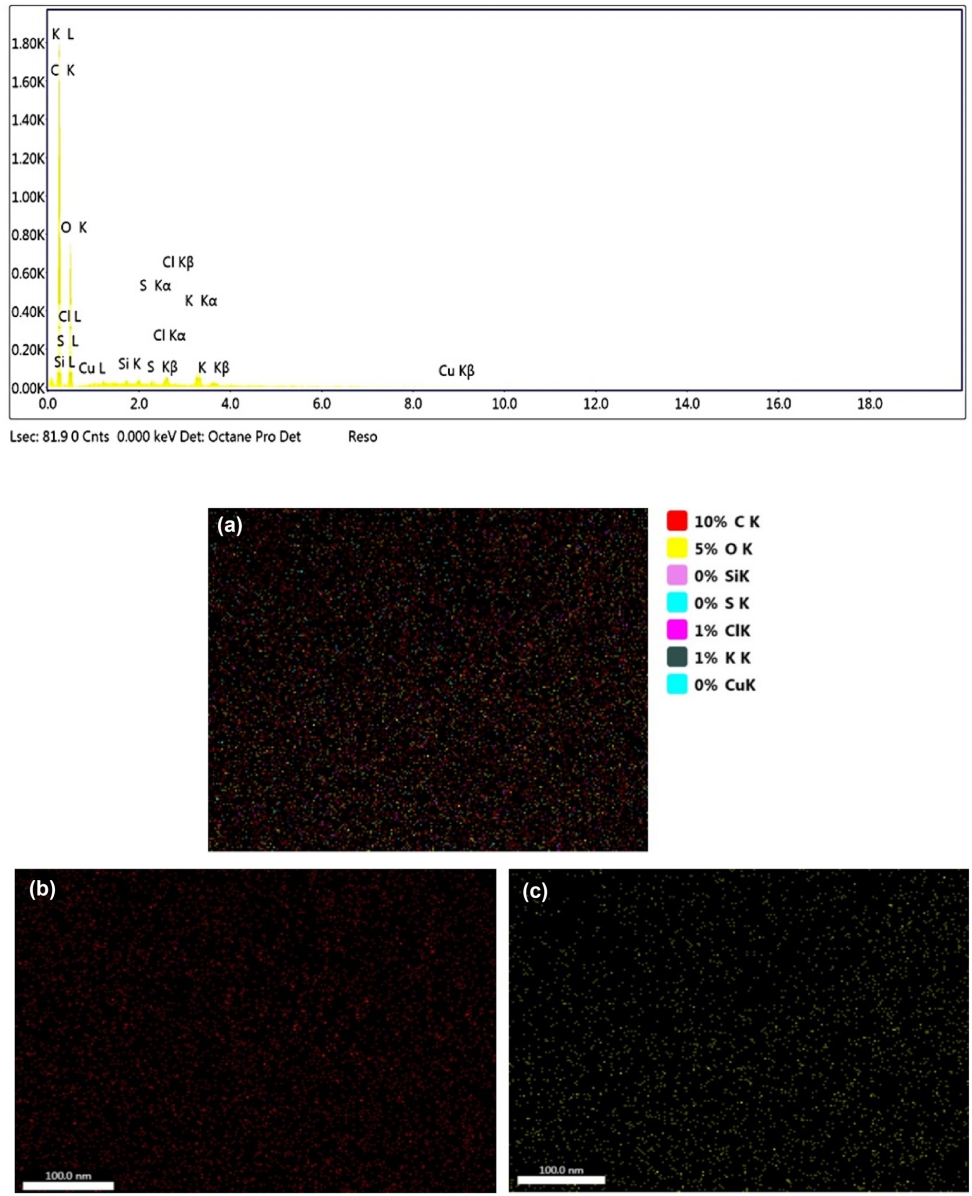

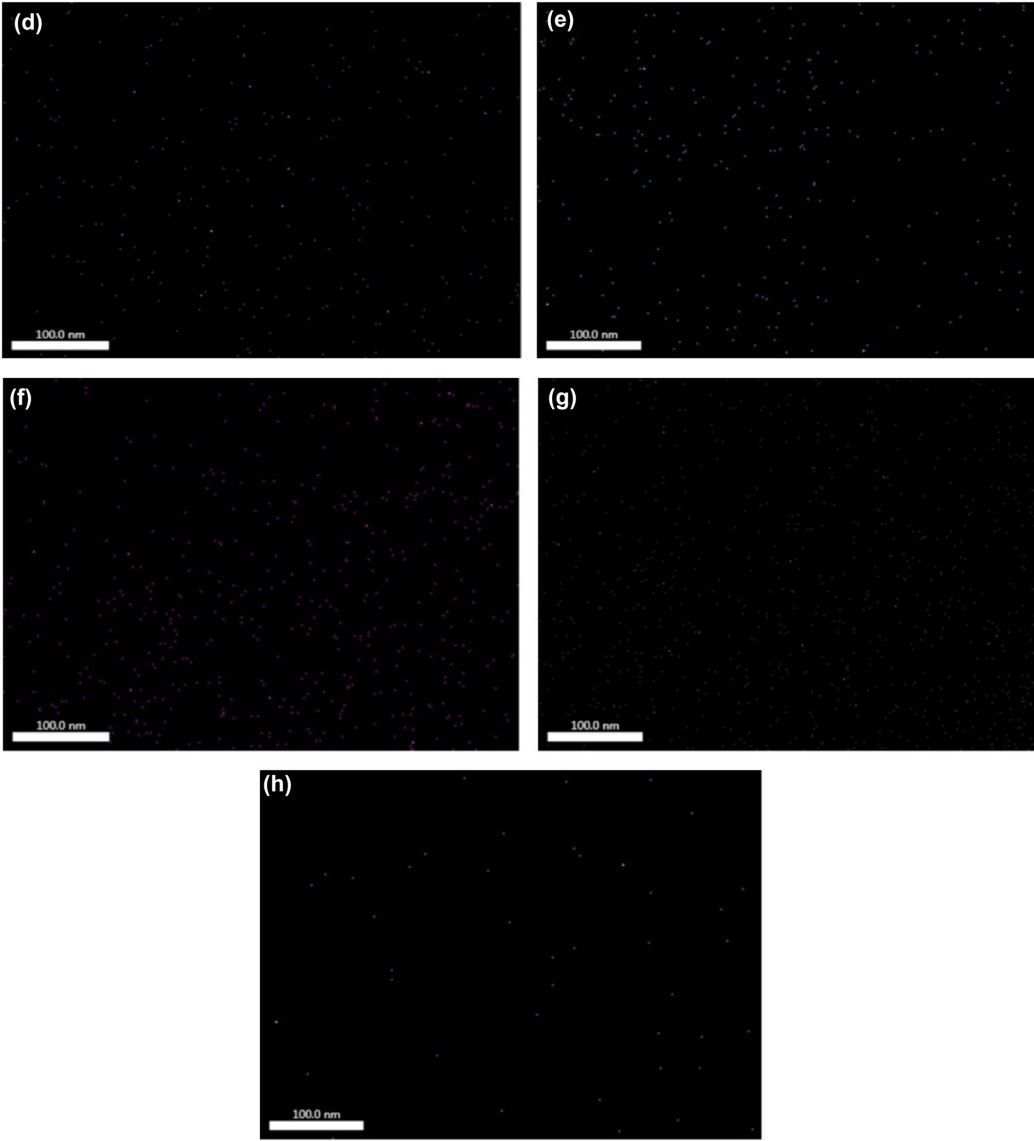
EDS elemental analysis scan of the archaeobotanical material; (**a**) Percentage distribution of all the identified elements; (**b**) C element; (**c**) O element; (**d**) Si element; (**e**) S element; (**f**) Cl element; (**g**) K element; (**h**) Cu element.

The microchemical analysis results of the archaeobotanical specimen are given in Table 1. The elemental mapping show that C and O are homogenously distributed with weight percentages of 55.76 and 41.16%, respectively, while Si, S, Cl, K and Cu are found distributed in cell walls and voids. The inorganic elements are distributed along the outer layer of cone (scaley skin) towards the ovules in the scanned area (Fig. 5).

**Table 1:** Elemental analysis results of the archaeobotanical material.

Element	Weight %	Atomic %	Net Int	Net Int. Error
C K	55.76	63.63	86.8	0.01
O K	41.16	35.26	45.3	0.02
Si K	0.2	0.1	1.7	0.35
S K	0.25	0.11	2.1	0.25
Cl K	0.74	0.29	5.5	0.16
K K	1.59	0.56	9.9	0.07
Cu K	0.3	0.07	0.4	0.63

**Figure 6:**
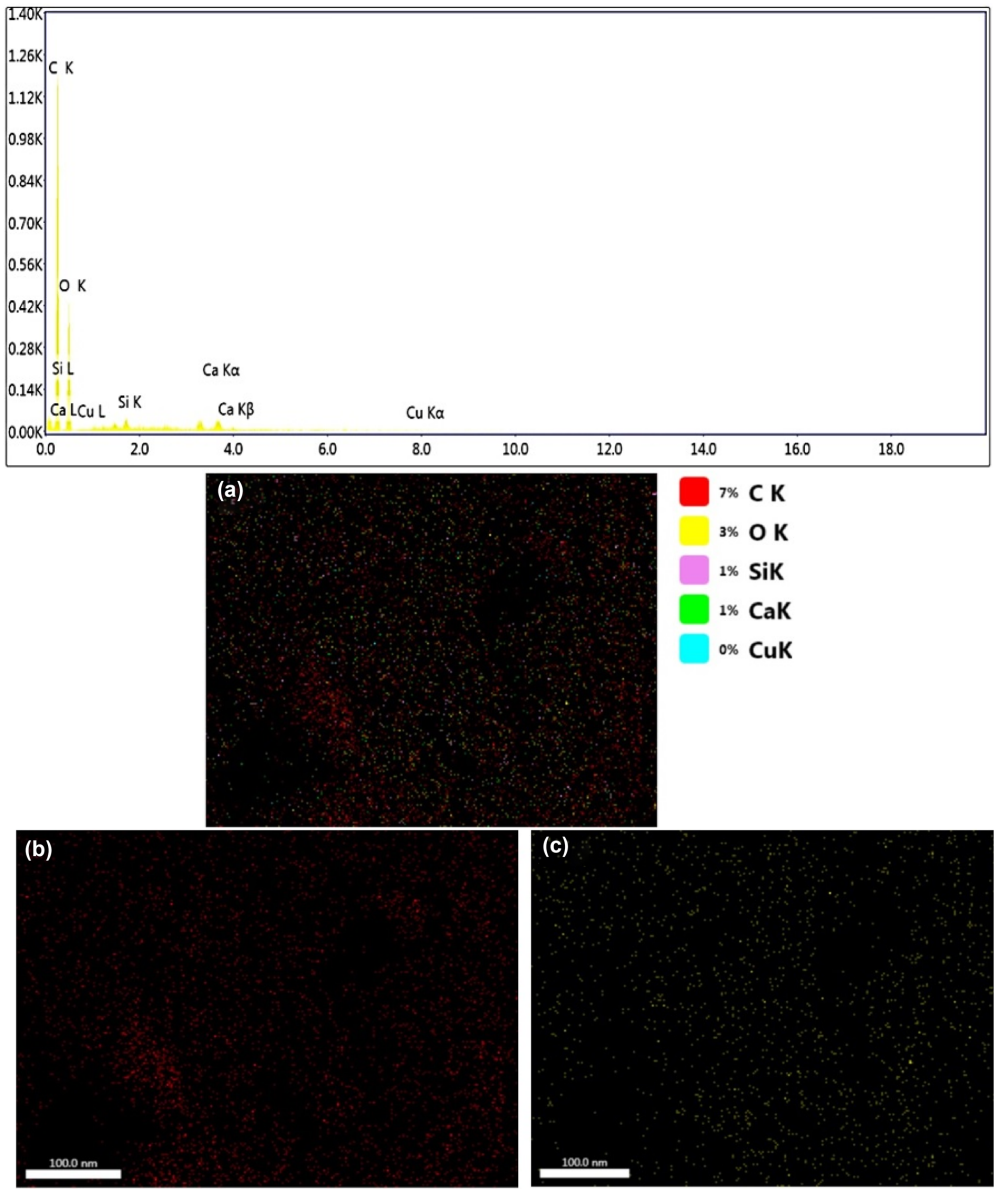

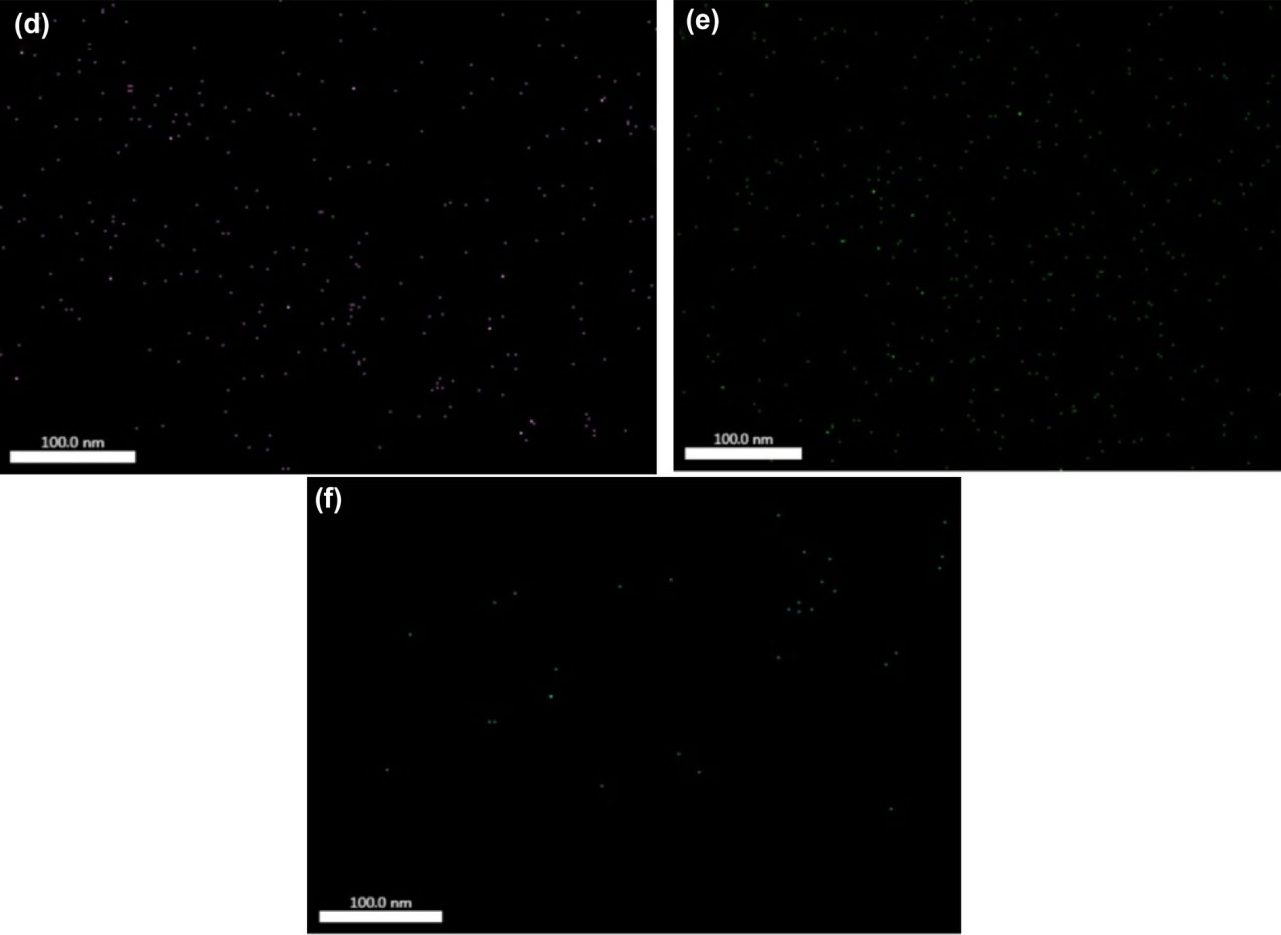
EDS elemental analysis scan of modern juniper. (**a**) Percentage distribution of all the identified elements; (**b**) C element; (**c**) O element; (**d**) Si element; (**e**) Ca element; (**f**) Cu element.

The analysis results of the modern cone specimen are given in Table 2. They indicated the presence of C and O, as main elemental composition with weight percentages of 59.93 and 38.6%, respectively, other elements such as Si, Ca and Cu were also identified (Fig. 6). The elements in both specimens almost followed the same manner in their distribution. However the archaeological specimen contains chloride, sulphur and copper. These elements are of the common components of copper corrosion.

**Table 2:** Elemental analysis results of a modern juniper cone.

Element	Weight %	Atomic %	Net Int	Net Int. error
C K	59.93	67.04	65.3	0.01
O K	38.6	32.42	24.9	0.02
Si K	0.38	0.18	2	0.19
Ca K	1.07	0.36	3.5	0.15
Cu K	0.02	0	0	1

## Discussion

Scanning electron microscopy proved to be efficient for studying small fossils, charcofied and lignified mesofossils^16^. Their assemblage shown well-preserved angiosperm seeds, flowers, fruits, leaf fragments, wood, shoots, cone scales, leaves, pollen cones, and seeds of conifers^16^. µ-computed tomography scanning (µCT) was also used to investigate fossil cones of *Pinus* sp. and *Keteleeria* sp.^17^.

X-ray micro-computed or computed tomography (CT) is ideal for studying three-dimensional fossils, it can be a good tool for the identification and the documentation of seed cones and other part of plants^18–23^. It was used to study permineralized plant fossils^24^ and to identify fruits and seeds in pyrite-permineralized specimens from the London Clay Formation^25^. Other methods such as diffuse X-ray methods have also been applied for investigating fossil fruits, e.g., *Crepetocarpon*^26^ and *Spirematospermum*^27^.

By comparing the archaeobotanical material with the modern taxa using visual, CT and SEM investigations, it was found that the archaeobotanical specimen can be identified as seed cones. They show a close affinity with juniper sp. Juniper cones have been found in ancient Egyptian tombs in multiple locations and were studied by visualizing morphology as fleshy berry-like cones^28,29^.

Cones of *Juniperus excelsa* and *J. oxycedrus* were found also in the tomb of Tutankhamen (1341–1323 BC)^30^. Medicinal use of juniper cone goes back to ancient Egypt 1500 BC, it was mentioned in a prescription for treating a tapeworm infection and for mummification^31,32^ and from ancient times it has been widely used as herbal medicine as antidiarrhoeal, anti-inflammatory, astringent, and antiseptic^33,34^. Volatile juniper seed oil was also used as a laxative^35^. The Romans used juniper for treatment of stomach diseases, as well as a cheap domestically-produced substitute for the expensive black pepper^36–38^. The Greeks also used juniper as medicine and more interestingly, on many of their Olympic Games occasions due to their belief that juniper cones increase the physical endurance of athletes^39^.

*J. phoenicea* was reported and conserved in northern mountains of Sinai^40,41^, although, *Juniperus* sp. are not known to grow in Egypt^30^. They were most probably imported from Greece^42^. Earlier studies showed that *J. oxycedrus*subsp. *oxycedrus*^43^ populated in East- and West-Mediterranean origins.

Based on morphological data, *J. excelsa* is divided into two subspecies^44,45^: *J. excels subsp. excelsa*, covering mountain and sub-mountain areas from the Balkan Peninsula in the west, through Anatolia, Syria and Lebanon to Crimea in the north and Iran in the east^44–47^, and *J. excels subsp. polycarpos* (K. Koch) Takht., found further to the east with a Transcaucasian-Central-Asian distribution. Based on random amplified polymorphic DNA (RAPD) molecular markers, these are considered as two taxa of separate species, *J. excelsa* and *J. polycarpos*, respectively^48^.

Seeds in *J. excelsa* subsp. *polycarpos* are (2-)3–6(-8) per cone^49^. Cones containing 1–13 seeds from 14 *J. excelsa* populations sampled from Greece, Cyprus, Ukraine, Turkey and Lebanon^50^, where 13 seeds/cone were found from Greece and Turkey trees. Other study, observed that seeds number/per cone of *J. excelsa* ranged between 1–7, 2–8, and 1–7 for Ziarat, ZarghoonGhar and Harboi provenances in Balochistan, respectively^51^.

*J. oxycedrus* subsp. *oxycedrus* with reddish-brown cones about 1 cm across had recorded the number of seeds in a female cones with two whorls of ovules with 3–6^52^, as described for *J. communis* by Schulz et al.^53^. Female cones with four and six ovules have been found in *J. oxycedrus* subsp. *oxycedrus* and in *J. communis*^53,54^. The abnormal type of female cone showed less than three seeds in one particular cone which has developed a restricted number of ovules, and the lack of success of pollination and fertilization of a normal 3-ovule cone^55^.

*J. communis* is a very variable species with differences in morphology and habitat over an extensive circum-polar geographical range, with five^44^ or seven^48^ varieties. The female cones may be dry and woody (e.g. *Cupressus*) or succulent (e.g. *Juniperus, Thuja*) and have cone scales arranged in opposite pairs or in threes with one to many ovules^56^. Seeds number of *J. communis* per cone and the filled seeds number per cone varied significantly between geographical regions and among populations within regions^57,58^. The cone containing 1–3, rarely 4, seeds per cone of *J. communis* distribution range in Europe collected for 31 populations/seven distinct regions^57^. Through 4000 ripe seed cones from 50 J*. communis* shrubs collected randomly (60–100 cones per shrub), the seeds number per cone were ranged from 1to 5^59^. Female strobili normally contain three ovules and thus produce 1–3 seeds (although up to 6 is possible)^60^. Seed cones of common juniper usually have three seeds (varying 1–6)^56^. Filled seeds of *J. communis* contain a well-developed, firm, off-white (sometimes brownish) embryo and megagametophyte and, therefore, are scored as probably viable, while the empty seeds are entirely empty, contain shrivelled contents, or are embryo-less and, therefore, are scored as nonviable^61^. With more than 95%, the ripe cones contain seeds number up to 3, while 5% produced from 4 to 5 seeds per cone from *J. communis* shrubs collected from Mishu-Dagh Altitudes in North West of Iran^59^. Ripe cone production correlated positively to seed set and seed predation but was independent of the percentages of empty and filled seeds^62^. The loss of seed per cone were due to predispersal seed predation and the abortion of seed^57^. Also, it was observed that populations from the Mediterranean mountains (south-east Spain) has the highest values in the seeds number/cone but the lowest values in the number of filled seeds per cone^57^. Furthermore, the proportion of three-seeded cones was greater in the open than under forest shade (43.8% and 2.6%, respectively)^63^. *J. communis* L. subsp. *communis* produces a large proportion of empty seeds. From the literature, the ripen cones are usually mentioned with empty places with no seeds. But it turns out that there are five chambers, but only three seeds^61,64,65^. Also, the seed quality of *J. communis* can be affected by the environment or forest ecology and nutrient, where the lower seed productivity can be a result of limited pollen availability or lower pollen quality and pollen growth rates^66–68^. In contrast, the opposite was observed in plants growing in nutrient-poor environments^67^. Seeds number of the *J. communis* berries are dependant on seed quality that can be observed when cutting the berries. Through visual assessment of some vesicles filled and other vesicles empty seeds (entirely empty) or shrivelled contents, or embryo-less, the number in the majority of cones is five vesicles but not all of them contain the seeds^61^.

We propose some possibilities for the use of the archaeological juniper cones in focus of the context; being buried with utensils, they could be used for the preparation of medical prescriptions, or they could be used for the flavoring of food. The other possibility is related to the wrestlers' statuette and the juniper intake by athletes to increase endurance.

In order to explain how and why the archaeobotanical materials have survived and preserved in an excellent conditions (Fig. 2c), for more than 2 millenniums and how they opposed both abiotic and biotic degradation. The location of the excavation site and soil condition were taken into consideration. Plant fossils are generally preserved in environments that are very low in oxygen (*e.g.,* anaerobic sediment) as most decomposers (e.g., fungi and some bacteria) require oxygen for metabolism. Plant fossils are commonly preserved in fine-grained sediment such as sand, silt, or clay. Organic material may also be protected in fine textured clay soils than coarse sandy ones. Silt is the main constituent of soil texture that leads to poor drainage and a significant water holding capacity due to its texture taking into account the depth of buried metals and water level on the site^69^. The silt soil of Sais site, where the archaeobotanical materials were found, was advantageous for the preservation process. The rate of biological degradation of organic materials in soil was also affected by their molecular structure, while cellulose is consumed preferentially over lignin and other poly-phenols present in plant. Both organic and inorganic matters are degraded in burial environments. Long-term burial changed the appearance and the chemical nature of the buried metal objects, resulting in the formation of corrosion of metals, and in some cases the complete destruction of the artefacts^70,71^. There are different parameters, which affect the corrosion process, *i.e.,* the metallurgy of the artefacts and the characteristics of the burial soil^72–74^. Soluble anions such as Cl^-^ and SO4^–^ in high amount in burial environment cause severe corrosion in the long-term. In fact, the presence of high amount of soluble salt results in increasing conductivity of the soil and accelerating electrochemical reactions leading to corrosion of archaeological copper alloys^71^. The presence of soluble sulphate may due to the presence of calcium sulphate phases in the composition of soil because of gypsum used as a binder or plaster in the architecture. The presence of sulphide (metallic sulphide) and its oxidation forms sulphuric acid, acidifies the soil and decreases pH^75^. Increasing acidity reduces organic degradation. In addition to the amount of corrosive anions in the soil, pH, the concentration of soluble salts and texture of the soil affect the preservation condition. Basic copper sulphates are stable in acidic conditions. By changing the pH of the environment to an alkaline condition, they will transform to more stable compounds^76^. These products will transform into green coppertrihydroxychlorides (basic copper chlorides) in the presence of high concentration of soluble chloride ions^77,78^. This product is responsible for the green hue of the archaeobotanical specimens. The antimicrobial effect of copper has been known for centuries^79–81^, so the presence of copper fragments in the find played a role in preventing organic decomposition.

From the microanalysis results and mapping of elements distribution, it was found that silica and copper precipitated in cell walls (Fig. 6), while chloride precipitated in the cell voids. Mineralization of plants by metals has previously been recorded^15^. This usually occurs when minerals carried in solution (silica, carbonate, chloride, etc.,) are deposited around plant cell surfaces or in the cell wall and intercellular spaces, encasing the plant structure^14,82^ and called structural preservation. The presence of a hard coat and antioxidants in the plant are also possible causes of good preservation^83^. The initial silica deposition begins within cell walls rather than in the cell lumina. The initial silica precipitation involves the affinity of silicic acid for hydroxylgroups in hollocelluloses and lignin. This phenomenon was also observed in the studied specimens. The silicification sequence in early stages is called “organic templating”^84–86^. It can be concluded that there have been many factors affected the preservation condition of the archaeobotanical material, resulting in initial stages of fossilization and mineralization. The unique preservation mode is greatly enhanced by the presence of metal fragments in addition to burial environment.

## Conclusion

In this study, unknown archaeobotanical materials from Sais archaeological site in Egypt, were identified. They show similar cone shapes and anatomical features of (*Juniperus* sp.). CT- Scanning and SEM–EDS investigations were used for detailed comparison with modern juniper cones. The archaeobotanical cones composed of five rounded to oval seeds in cone shaped 0.8–1.3 cm diameter. The unique preservation condition is discussed as regards the burial environment; the kind and texture of soil, soluble anions such as Cl^-^ and SO4^–^, pH and the presence of metals.
